# Clinical correlates of depressed adolescents comorbid with food addiction: a cross-sectional study

**DOI:** 10.3389/fpsyt.2026.1909728

**Published:** 2026-08-19

**Authors:** Liling Sun, Lewei Liu, Pei Tang, Haojie Fan, Mingru Hao, Kai Zhang, Huanzhong Liu

**Affiliations:** Department of Psychiatry, The Fourth Affiliated Hospital of Anhui Medical University, Hefei, Anhui, China

**Keywords:** adolescent, clinical characteristics, food addiction, major depressive disorder, receiver operating characteristic

## Abstract

**Background:**

Food addiction (FA) involves the excessive intake of ultra-processed foods and is prevalent among individuals with major depressive disorder (MDD). Despite this association, evidence regarding FA in adolescents with MDD is limited. This investigation sought to examine the relationship between FA and clinical features in this demographic, aiming to clarify the prevalence of FA and offer theoretical insights for clinical management.

**Methods:**

A total of 194 depressed adolescents were recruited at the Fourth Affiliated Hospital of Anhui Medical University between September 2022 and June 2025. Validated multidimensional scales were used to assess FA, depression, anxiety, perceived stress, and internet addiction (IA). Subsequently, stepwise regression analysis was performed to identify independent factors of FA, and receiver operating characteristic (ROC) curve analysis was conducted for these predictors, with the area under the curve (AUC) calculated.

**Results:**

The prevalence of FA in depressed adolescents was 17.5%. Multivariate logistic regression identified BMI (OR = 1.149, 95% CI = 1.051 - 1.257, *P* = 0.002), anxiety (OR = 1.116, 95% CI = 1.039 - 1.198, *P* = 0.002), perceived stress (OR = 1.137, 95% CI = 1.030 - 1.254, *P* = 0.011), and IA (OR = 1.051, 95% CI = 1.020 - 1.083, *P* = 0.001) as independent risk factors of FA. Furthermore, ROC curve analysis demonstrated that a combined model comprising these four factors exhibited superior discriminatory ability for identifying FA (AUC = 0.861, 95% CI = 0.801 - 0.921, *P* < 0.001).

**Conclusion:**

FA affects some adolescents with MDD and is closely associated with BMI, anxiety, perceived stress, and IA. Clinicians should integrate screening for this abnormal eating behavior and related psychosocial factors into routine care.

## 1 Introduction

Major depressive disorder (MDD), a primary contributor to global disability, is anticipated to become the leading cause of disease burden worldwide by 2030 (1). Influenced by societal changes and escalating life stressors, the onset of MDD is increasingly occurring at younger ages, making it the most common emotional disorder among adolescents (2). Appetite disturbance is a core symptom of MDD, with specific manifestations varying across subtypes (3). Typical depression is characterized by reduced appetite and weight loss, whereas atypical depression is marked by hyperphagia (increased appetite) and weight gain. Previous research and clinical practice have predominantly focused on symptoms of appetite reduction, often overlooking maladaptive eating behaviors associated with appetite increase. However, a growing body of literature suggests that patients with MDD exhibit a significantly elevated likelihood of engaging in maladaptive eating behaviors characterized by increased appetite compared to the general population (4, 5). Adolescence represents a critical window for the emergence of such maladaptive eating behaviors (6). Given the incomplete development of cognitive and psychological faculties, as well as limited ability to regulate emotions and impulses, adolescents suffering from MDD exhibit a pronounced predisposition toward these behaviors (7). The co-occurrence of maladaptive eating behaviors not only complicates the treatment of MDD but also exerts profound negative impacts on patients’ prognosis, physical growth and development, and social functioning.

Food addiction (FA) is an emerging concept used to understand maladaptive eating behaviors, characterized by compulsive consumption of highly palatable foods (high in sugar, fat, or carbohydrates) and accompanied by withdrawal symptoms such as anxiety and depression upon cessation (8, 9). The most prevalent symptoms of FA include intense cravings for and loss of control over the intake of certain ultra-processed foods (10, 11). Currently, there is no complete consensus within the academic community regarding the conceptual definition of FA (12). Some scholars argue that food, as a necessity for survival, is fundamentally distinct from traditional addictive substances; furthermore, the Diagnostic and Statistical Manual of Mental Disorders, Fifth Edition (DSM-5), does not currently list FA as an independent diagnostic category of mental disorder. However, opposing viewpoints suggest that modern ultra-processed foods differ significantly in composition from natural foods, and that FA may actually stem from dependence on food additives, which increase the risk of excessive consumption of specific food items (13). Therefore, attention to this eating behavior in clinical practice is of significant importance. Existing epidemiological studies on FA have predominantly focused on the general healthy population, with relatively scarce research involving patients with MDD. For instance, Chen et al. reported a FA prevalence rate of 9.2% among students at two normal universities in Hunan Province, China (14); however, this sample did not focus on clinical patient populations. Given the current gap in research concerning adolescents with MDD, further exploration of the prevalence and psychosocial mechanisms underlying FA in this population holds substantial clinical value for early screening, preventive intervention, and the optimization of treatment strategies.

Previous clinical studies have indicated that the psychosocial factors influencing FA in patients with MDD are multidimensional. First, sociodemographic characteristics, such as gender, age, body mass index (BMI), and educational attainment, may be closely associated with FA. For instance, Mills et al. found that female patients with MDD scored higher on scales measuring restrained and emotional eating compared to their male counterparts, and a significantly greater proportion met the diagnostic criteria for FA (15). Furthermore, a meta-analysis demonstrated a significant positive correlation between weight status and FA severity among children and adolescents (16). Second, unhealthy lifestyles constitute potential influencing factors. Two recent surveys conducted among general university student populations indicated that mobile phone addiction is positively correlated with the frequent consumption of fast food, carbonated beverages, and other sugar-sweetened foods (17, 18). This suggests that digital behavioral dependence may influence FA risk by altering dietary patterns. Regarding psychopathological mechanisms, depressive and anxious emotions directly regulate dietary behaviors in adolescents. Evidence indicates that depressive mood can affect individual food choices by altering appetite regulation mechanisms or food preferences (19). A substantial body of research has demonstrated a significant link between the intensity of anxiety and depressive symptoms and various dysregulated eating patterns, such as restrained, emotional, and external eating, as well FA (4, 19–21). Moreover, stress is a critical factor in the onset and development of FA. Individuals often rely on the euphoria induced by highly palatable foods to alleviate anxiety in stressful situations; this maladaptive coping strategy leads to increased intake of high-sugar and high-fat foods, thereby exacerbating the risk of FA (22). Lim et al. further highlighted through longitudinal research that the interaction between anxiety and stress during adolescence can predict the future risk of binge eating (23). Based on the aforementioned studies, numerous psychosocial factors likely contribute to the occurrence of FA. However, research directly focusing on adolescents with MDD remains insufficient. Given the unique characteristics of this population, clarifying the specific mechanistic links between these factors and FA represents a critical research gap that urgently needs to be addressed.

Therefore, the primary objectives of this research were to determine the prevalence of FA among adolescents with MDD and analyzed its associations with demographic characteristics and psychosocial factors. The findings will be intended to underpin the development of targeted early detection, therapeutic, and preventive strategies targeting FA in this population.

## 2 Methods

### 2.1 Participants

This cross-sectional study was conducted at the Department of Psychiatry (outpatient and inpatient units) of the Fourth Affiliated Hospital of Anhui Medical University between September 2022 and June 2025. The study aimed to recruit adolescents with MDD as participants, all of whom underwent rigorous screening. Inclusion criteria: (1) Age between 12 and 18 years; (2) Primary diagnosis of MDD based on DSM-5 criteria; (3) Absence of any comorbid eating disorders (including anorexia nervosa, bulimia nervosa, and binge-eating disorder) as defined by DSM-5. Exclusion criteria: (1) Currently or previously diagnosed with other serious mental disorders, such as schizophrenia, substance use disorder, etc.; (2) Presence of severe medical comorbidities, such as acute/chronic infections, autoimmune diseases, or other serious somatic illnesses; (3) Use of corticosteroids, immunomodulators, or other medications that could significantly affect appetite within the past year; (4) Inability to complete the prescribed assessment procedures due to impaired cognitive function, poor cooperation, or other reasons. The study protocol was reviewed and approved by the Ethics Committee of the Fourth Affiliated Hospital of Anhui Medical University (Approval No.: KYXM-202210-014). Prior to study initiation, researchers provided detailed information regarding the study’s objectives, procedures, and potential risks to all potential participants and their legal guardians. Written informed consent was obtained after ensuring full understanding of the study details.

### 2.2 Measuring instruments

#### 2.2.1 General data

General data for all adolescent patients were collected using a self-designed questionnaire. The collected variables included: gender (male = 1, female = 2), age (years), BMI, educational level (junior high school or below = 1, senior high school or above = 2), school location (urban = 1, rural = 2), age at onset (years), duration of illness (months), and antidepressant medication usage [none = 1, selective serotonin reuptake inhibitors (SSRIs) = 2, others = 3].

#### 2.2.2 FA

The Yale Food Addiction Scale (YFAS) was employed to assess participants’ tendencies toward FA (24). The scale comprises 25 items and evaluates FA based on the following eight criteria: (1) intake exceeding intended amounts or duration; (2) chronic desire or failed efforts to restrict intake; (3) substantial time investment in acquiring, consuming, or recovering from food; (4) relinquishment of key social, occupational, or leisure activities; (5) persistence in consumption despite awareness of physical or psychological harm; (6) evidence of tolerance; (7) experience of withdrawal symptoms; and (8) presence of clinically significant impairment or distress. For the first seven criteria, a “yes” response was scored as 1 and a “no” as 0, with the sum yielding a symptom count ranging from 0 to 7. The YFAS assesses FA using two methods: first, by evaluating the tendency toward FA via the symptom count; and second, by establishing a diagnosis of FA, which requires a symptom count of ≥3 along with meeting the eighth criterion. The YFAS is currently a widely recognized instrument for predicting and diagnosing FA, and its Chinese version has been preliminarily applied among Chinese adolescents, demonstrating good reliability and validity (14, 25).

#### 2.2.3 Depressive symptoms

Depressive symptoms over the past week were assessed using the 17-item Hamilton Depression Rating Scale (HAMD-17) (26). The scale comprises 17 items, with total scores ranging from 0 to 52; higher scores indicate more severe clinical depressive symptoms. The HAMD-17 demonstrates good reliability and validity and has been widely used to assess depression severity in adolescent patients (27).

#### 2.2.4 Anxiety symptoms

Anxiety symptoms over the past week were evaluated using the Hamilton Anxiety Rating Scale (HAMA) (28). This scale consists of 14 items, each rated on a 5-point Likert scale ranging from 0 (no symptoms) to 4 (most severe). Total scores range from 0 to 56, with higher scores indicating greater severity of clinical anxiety symptoms.

#### 2.2.5 Perceived stress

Perceived stress levels were measured using the Perceived Stress Scale (PSS) (29). The PSS assesses the degree to which individuals perceive situations in their lives as unpredictable, uncontrollable, or overloaded. It contains 10 items, each scored on a 5-point scale, with total scores ranging from 0 to 40. Higher scores reflect higher levels of perceived stress.

#### 2.2.6 Internet addiction

IA was assessed using the Internet Addiction Test (IAT) (30). The IAT comprises 20 items addressing internet usage frequency and related scenarios. Each item is scored from 1 to 5, resulting in a total score range of 20 to 100. Higher scores indicate a greater severity of internet addiction. The Chinese version of the IAT has been fully validated among Chinese adolescents (31).

### 2.3 Statistical analyses

Statistical analyses were conducted using IBM SPSS software (version 23.0). The Kolmogorov-Smirnov test was employed to assess the normality of continuous variables. Data following a normal distribution are reported as mean ± standard deviation (SD) and were compared using independent-samples t-tests. For non-normally distributed data, values are presented as median and interquartile range [M (P_25_, P_75_)], with differences analyzed via the Mann-Whitney U test. Categorical variables are expressed as counts and percentages [n (%)], and comparisons between groups were performed using the chi-square (χ^2^) test. Pearson or Spearman correlation analyses were performed to assess the associations between variables. In the multivariable analysis phase, given the relatively large number of candidate covariates coupled with a limited sample size, and the current lack of clear theoretical consensus regarding adjustment strategies for confounders of FA, this study employed stepwise logistic regression (for binary outcomes) and stepwise linear regression. This approach was adopted to balance model fit and complexity, aiming to identify an optimal and parsimonious set of predictors significantly associated with FA from among the numerous candidate variables. Candidate variables included sex, age, antidepressant medication class, and variables with *P* < 0.05 in the univariate analysis. Prior to multivariable analysis, multicollinearity among variables was assessed by calculating the variance inflation factor (VIF). The results showed that all VIF values were less than 5.0, well below the recommended threshold of 10, indicating no evidence of multicollinearity in this study. Additionally, receiver operating characteristic (ROC) curve analysis was conducted, and the area under the curve (AUC) was calculated to evaluate the discriminatory ability of each independent risk factor for FA (binary classification). A *P* value < 0.05 was considered statistically significant.

## 3 Results

### 3.1 Comparison of sociodemographic data and clinical features between FA and non-FA groups

A total of 194 adolescents with MDD were enrolled in this study, of whom 64 were male (33.0%), with a mean age of (15.21 ± 1.63) years. Regarding clinical characteristics, the mean age at first onset was (13.64 ± 1.86) years, mean duration of illness was (18.82 ± 14.91) months, mean HAMD-17 score was (20.27 ± 5.14), mean HAMA score was (21.93 ± 7.22), mean PSS score was (26.27 ± 5.67), and mean IAT score was (52.85 ± 15.15). Univariate analysis showed that, compared with the non-FA group, the FA group had higher BMI (t = -2.823, *P* = 0.008), longer duration of illness (Z = -2.095, *P* = 0.036), higher HAMD-17 scores (t = -3.918, *P* < 0.001), higher HAMA scores (t = -4.213, *P* < 0.001), higher PSS scores (t = -4.890, *P* < 0.001), and higher IAT scores (t = -4.617, *P* < 0.001) (**Table 1**).

**Table 1:** Comparison of demographic information and clinical features between FA and non-FA groups.

Variables	Total sample (N = 194)	FA (N = 34)	Non-FA (N = 160)	t/Z/χ^2^	*P*
Demographic information					
Gender, n (%)				2.868	0.090
Males	64 (32.99)	7 (20.59)	57 (35.62)		
Females	130 (67.01)	27 (79.41)	103 (64.38)		
Age (years), mean (SD)	15.21 (1.63)	15.32 (1.63)	15.19 (1.63)	-0.442	0.659
Education, n (%)				0.004	0.953
Junior high school and below	79 (40.72)	14 (41.18)	65 (40.63)		
Senior high school and above	115 (59.28)	20 (58.82)	95 (59.37)		
School location, n (%)				0.733	0.392
Urban	166 (85.57)	27 (79.41)	139 (86.88)		
Rural	28 (14.43)	7 (20.59)	21 (13.12)		
BMI (kg/m^2^), mean (SD)	21.60 (4.70)	24.34 (6.61)	21.02 (3.97)	-2.823	0.008
Age at onset (years), mean (SD)	13.64 (1.86)	13.35(1.63)	13.70 (1.91)	0.986	0.325
Duration of illness (months), median (P_25_, P_75_)	12.00 (6.00, 27.00)	24.00 (11.50, 36.00)	12.00 (6.00, 24.00)	-2.095 ^a^	0.036
Antidepressants, n (%)				2.883	0.237
None	49 (25.26)	8 (23.53)	41 (25.63)		
SSRIs	126 (64.95)	20 (58.82)	106 (66.25)		
Others	19 (9.79)	6 (17.65)	13 (8.12)		
Clinical features					
HAMD-17 score, mean (SD)	20.27 (5.14)	23.29 (3.91)	19.63 (5.15)	-3.918	<0.001
HAMA score, mean (SD)	21.93 (7.22)	26.47 (5.98)	20.96 (7.11)	-4.213	<0.001
PSS score, mean (SD)	26.27 (5.67)	30.35 (4.80)	25.41 (5.47)	-4.890	<0.001
IAT score, mean (SD)	52.85 (15.15)	63.21(15.14)	50.64 (14.25)	-4.617	<0.001

FA, Food Addiction; BMI, Body Mass Index; SSRIs, Selective Serotonin Reuptake Inhibitors; HAMD-17, 17-item Hamilton Depression Rating Scale; HAMA, Hamilton Anxiety Rating Scale; PSS, Perceived Stress Scale; IAT, Internet Addiction Test; ^a^Mann-Whitney U test; SD, Standard Deviation; Bolded *P* value: < 0.05.

### 3.2 Correlation analysis of the symptom count of FA with sociodemographic data and clinical data

Correlation analysis showed that among adolescents with MDD, a higher number of FA symptoms was associated with educational attainment of junior high school and below and attending a rural school (all *P* < 0.05). In addition, the number of FA symptoms was positively correlated with HAMD-17, HAMA, PSS, and IAT scores (all *P* < 0.05) (**Table 2**).

**Table 2:** Correlation analysis of the symptom count of food addiction with general demographic data and clinical data.

Variables	r	*P*
Gender	-0.091	0.208
Age (years)	-0.122	0.090
Education	-0.169	0.019
School location	0.161	0.026
BMI (kg/m^2^)	0.075	0.297
Age at onset (years)	-0.090	0.211
Duration of illness (months)	-0.008	0.913
Antidepressants	0.050	0.489
HAMD-17 score	0.257	<0.001
HAMA score	0.319	<0.001
PSS score	0.372	<0.001
IAT score	0.446	<0.001

BMI, Body Mass Index; HAMD-17, 17-item Hamilton Depression Rating Scale; HAMA, Hamilton Anxiety Rating Scale; PSS, Perceived Stress Scale; IAT, Internet Addiction Test.

Bolded *P* value: < 0.05.

### 3.3 Multivariate stepwise regression analysis of FA

First, stepwise logistic regression analysis was performed with FA status (binary classification) as the dependent variable. The results showed that BMI (OR = 1.149, 95% CI = 1.051 - 1.257, *P* = 0.002), HAMA score (OR = 1.116, 95% CI = 1.039 - 1.198, *P* = 0.002), PSS score (OR = 1.137, 95% CI = 1.030 - 1.254, *P* = 0.011), and IAT score (OR = 1.051, 95% CI = 1.020 - 1.083, *P* = 0.001) were independently associated with FA in adolescents with MDD (**Table 3**).

**Table 3:** Multivariate logistic stepwise regression analysis^a^.

Variables	B	SE	Wald χ^2^	OR	95% CI	*P*
BMI	0.139	0.046	9.251	1.149	1.051 - 1.257	**0.002**
HAMA score	0.110	0.036	9.171	1.116	1.039 - 1.198	**0.002**
PSS score	0.128	0.050	6.498	1.137	1.030 - 1.254	**0.011**
IAT score	0.050	0.015	10.411	1.051	1.020 - 1.083	**0.001**

BMI, Body Mass Index; HAMA, Hamilton Anxiety Rating Scale; PSS, Perceived Stress Scale; IAT, Internet Addiction Test; SE, Standard Error; OR, Odds Ratio; CI, Confidence Interval; Bolded *P* value: < 0.05.

^a^ With FA (binary classification) as the dependent variable.

When stepwise multiple linear regression analysis was conducted with the count of FA symptoms as the dependent variable, HAMA score (beta = 0.199, t = 3.070, *P* = 0.002), PSS score (beta = 0.208, t = 3.127, *P* = 0.002), and IAT score (beta = 0.370, t = 5.906, *P* < 0.001) remained significant independent correlates (**Table 4**).

**Table 4:** Multivariate linear stepwise regression analysis^b^.

Variables	Beta	t	*P*
HAMA score	0.199	3.070	**0.002**
PSS score	0.208	3.127	**0.002**
IAT score	0.370	5.906	**<0.001**

HAMA, Hamilton Anxiety Rating Scale; PSS, Perceived Stress Scale; IAT, Internet Addiction Test; Bolded *P* value: < 0.05.

^b^ With the number of FA symptoms as the dependent variable.

### 3.4 The predictive value of each independent factor for FA

**Figure 1:**
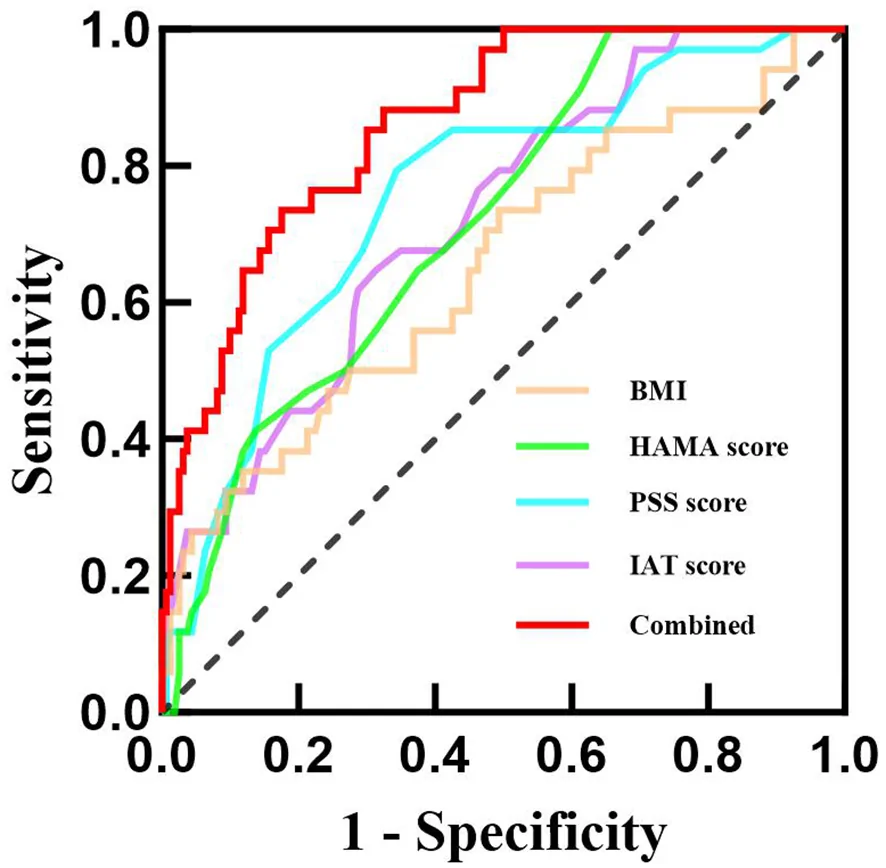
ROC curves for BMI, HAMA score, PSS score and IAT score predicting food addiction.

In this study, ROC curve analysis was performed for each independent variable identified in the multivariate logistic regression analysis to evaluate their discriminatory ability for FA. The results were as follows: BMI (AUC = 0.651, 95% CI = 0.544 - 0.758, *P* = 0.006); HAMA score (AUC = 0.717, 95% CI = 0.632 - 0.802, *P* < 0.001); PSS score (AUC = 0.756, 95% CI = 0.668 - 0.844, *P* < 0.001); IAT score (AUC = 0.723, 95% CI = 0.634 - 0.811, *P* < 0.001); and the combined four indicators (AUC = 0.861, 95% CI = 0.801 - 0.921, *P* < 0.001) (**Table 5**; **Figure 1**). These findings indicated that, compared with each individual predictor, the combined model of the four indicators demonstrated a larger AUC, superior discriminatory ability for FA, and achieved high sensitivity and specificity.

**Table 5:** Predictive value of each independent risk factor for FA.

Variables	Sensitivity	Specificity	AUC	95% CI	*P*
BMI	0.735	0.506	0.651	0.544 - 0.758	**0.006**
HAMA score	1.000	0.344	0.717	0.632 - 0.802	**<0.001**
PSS score	0.794	0.656	0.756	0.668 - 0.844	**<0.001**
IAT score	0.647	0.687	0.723	0.634 - 0.811	**<0.001**
Combined^**c**^	0.735	0.825	0.861	0.801 - 0.921	**<0.001**

FA, Food Addiction; BMI, Body Mass Index; HAMA, Hamilton Anxiety Rating Scale; PSS, Perceived Stress Scale; IAT, Internet Addiction Test; CI, Confidence Interval; Bolded *P* value: < 0.05.

^c^ Combination of BMI, HAMA score, PSS score and IAT score; AUC, Area Under the Curve.

## 4 Discussion

This study found that the prevalence of FA among adolescents with MDD was 17.5%, significantly higher than the 9.2% reported in healthy Chinese adolescents (14). However, it is imperative to interpret this prevalence estimate within the context of the ongoing scientific debate regarding the validity of FA as a distinct diagnostic construct. While the YFAS operationalizes FA based on substance use disorder criteria, critics argue that this framework may pathologize normative eating behaviors or conflate addiction with severe dietary restraint and obesity. Despite these conceptual limitations, the current finding aligns with existing literature treating FA as a clinically relevant phenotype. Currently, there is a paucity of research investigating the prevalence of FA specifically among children and adolescents with MDD. However, a recent study focusing on adolescents with psychiatric disorders reported a FA prevalence rate of 16.5% (32). Similarly, a Danish study found that symptoms of FA are widespread among adolescents with psychiatric disorders, particularly those with affective disorders and schizophrenia (33). Furthermore, studies conducted among adults with MDD have indicated that the prevalence of FA ranges from approximately 25% to 28% (4, 15). In summary, while acknowledging that FA remains a contested construct rather than a universally accepted diagnosis, our findings suggest a significantly elevated risk of FA-related symptoms among adolescents with MDD, underscoring the need for clinicians to pay heightened attention to this phenomenon as a potential marker of maladaptive coping or severe dysregulation, irrespective of its formal nosological status.

Regarding sociodemographic characteristics, this study found that depressed adolescents who met the criteria for FA had higher BMI. This finding was largely consistent with previous research conducted among adolescent and adult populations (33, 34). A recent systematic review of 15 studies across children, adolescents, and adults revealed that individuals with FA consumed higher amounts of energy, carbohydrates, and total fat, with specific associated foods including candies, baked sweets, salty snacks, fast food, and red or processed meats (35). Consistently, a domestic longitudinal cross-lagged study indicated that children and adolescents with FA face an increased risk of weight gain due to higher fat intake, with these adverse effects intensifying with age (36). Additionally, this study observed that a greater number of FA symptoms was associated with lower grade levels and rural school locations. suggesting that educational level and geographic setting may influence the occurrence of FA. These associations may reflect developmental and environmental factors: as educational attainment increases, brain development progresses, leading to improved inhibitory control (37), while adolescents’ increasing concern with body image may shift their interest from food to social activities (38). Adolescents with MDD living in rural areas may be more prone to developing unhealthy dietary habits due to lower quality of education and reduced parental supervision in these regions.

Regarding clinical manifestations, our results demonstrated a significant positive correlation between FA and the intensity of depressive, anxious, and stress-related symptoms. Regression analysis further revealed that anxiety and perceived stress levels are independently correlated with FA. Indeed, numerous previous studies involving diverse populations have reached similar conclusions (21, 39). For instance, a meta-analysis by Skinner et al., which included 27 clinical and non-clinical studies on adolescents, identified significant positive correlations between depression, anxiety, and FA (21). Another survey conducted among young people in Taiwan, China, also found that perceived stress levels are positively correlated with FA, and that FA plays a potential mediating role in the relationship between stress and BMI (40). Several mechanisms may underlie these associations. First, the “compensation theory” posits that individuals grappling with depression, anxiety, or elevated stress may resort to maladaptive coping strategies, such as consuming ultra-processed foods, to mitigate psychological distress; over time, this avoidance strategy may evolve into habitual excessive consumption (41). Additionally, diminished self-control associated with greater symptom severity can trigger impulsive behaviors, thereby increasing FA risk (42). From a biological perspective, the relationship between depressive, anxiety, and stress-related symptoms and FA may involve the hypothalamic-pituitary-adrenal (HPA) axis. Certain ultra-processed foods, particularly those high in artificial sugars and fats, may suppress physiological stress responses mediated by the HPA axis by reducing glucocorticoid sensitivity, thereby improving mood (43, 44). In essence, FA may function as a coping mechanism for depression, anxiety, and stress. Moreover, FA in adolescents with MDD may also be linked to dysregulation of dopamine levels. Previous research has reported that plasma dopamine levels are positively correlated with the severity of depression, anxiety, and stress-related symptoms (39). Elevated dopamine levels, in turn, activate reward systems associated with eating behavior (45). Nevertheless, these biological and psychological interpretations should be viewed through the lens of the aforementioned conceptual controversy. Notably, although univariate analysis indicated a significant association between depression severity and FA, this variable was not retained in the final model following stepwise regression. This suggests that after simultaneously controlling for anxiety, perceived stress, and other covariates, the independent explanatory power of depression for FA may be obscured by anxiety and stress. A potential mediation effect may exist among these three factors, whereby the impact of depression on FA is partially mediated indirectly through heightened levels of anxiety and stress rather than acting directly on the core pathological processes of FA. Future research urgently requires larger sample sizes, longitudinal designs, and structural equation modeling to clarify the temporal relationships and mediating pathways among depression, anxiety, perceived stress, and FA.

Interestingly, this study also identified a positive correlation between IA and FA, consistent with prior evidence linking problematic internet use to disordered eating and psychological distress (46). An online survey revealed significant associations among IA, psychological distress, and FA, suggesting that IA may exacerbate psychological distress and elevate the risk of FA by increasing body dissatisfaction and leading to the internalization of weight-related stigma (46). Furthermore, a meta-analysis comprising 22 studies confirmed that problematic internet use is a predictor of eating disorders in children and adolescents, highlighting the necessity for early prevention (47). The close relationship between IA and FA may be associated with specific clinical characteristics of depressed adolescents with IA, such as difficulties in impulse control and low levels of physical activity (48). Additionally, neuroimaging studies have indicated that FA and IA may share similar brain network mechanisms, specifically those involving dopamine-mediated reward circuits (49). Weinstein et al. pointed out that internet gaming disorder involves volumetric changes in the ventral striatum, a critical component of the brain’s reward system (50). Given the previously elucidated close relationship between FA and the reward system, it is understandable why FA is significantly correlated with IA. A case-control study conducted in China on patients with internet gaming disorder further confirmed that, compared to controls, patients exhibited deficits in reward regulation related to cravings for both gaming and food (51). However, it should be noted that this study did not assess these neural correlates, and thus we cannot directly confirm whether they serve as explanatory factors for the observed associations. Therefore, such interpretations remain speculative in nature and should be interpreted with caution pending empirical validation. Collectively, these studies suggest that the co-occurrence of FA and IA is prevalent. Given the high detection rate of IA among adolescents with MDD (52), clinicians should pay close attention to the potential risk of FA in this population and actively implement appropriate interventions. A descriptive study in Singapore noted that excessive use of the internet and mobile applications is associated with the severity of eating disorders; therefore, interventions targeting digital media usage should be integrated into the treatment of eating disorders (53). Consequently, future interventions could leverage internet platforms and mobile applications to educate adolescents with MDD about healthy dietary patterns and the hazards of unhealthy eating, thereby improving their dietary behaviors. Finally, to explore the discriminatory ability of various risk factors for FA, this study conducted a ROC curve analysis. The results indicated that the combination of BMI, HAMA score, PSS score, and IAT score had high discriminatory ability for FA. However, the AUC value of 0.861 for this model should be interpreted with caution. Given that both model development and performance evaluation were conducted using the same dataset, and in the absence of internal validation as well as external cohort validation, there is a potential risk of overfitting, which may lead to an overestimation of the model’s true generalizability. Therefore, the robustness of this predictive model warrants further verification through large-scale, multicenter prospective studies.

Several limitations of this study warrant consideration. First, the reliance on a small, single-center sample may constrain the external validity of our findings, underscoring the necessity for large-scale, multicenter replication studies. Second, the cross-sectional nature of the data prevents the establishment of causal relationships; thus, longitudinal prospective cohorts are required to determine temporal precedence and verify these associations. Furthermore, the results derived from stepwise regression in this study should be interpreted with caution. Although this method is appropriate for the current exploratory analysis, its inherent instability may lead to biased parameter estimates and an increased risk of model overfitting. Therefore, the findings of this study should be regarded as preliminary evidence, underscoring the urgent need for future validation through larger sample sizes and more robust variable selection strategies. Notably, although antidepressant medication class was included as a candidate covariate in the stepwise regression model, it was ultimately excluded during the variable selection process. This suggests that after controlling for psychological symptoms such as anxiety and stress, the independent explanatory power of medication class for FA may be relatively limited. However, given that this study did not collect key pharmacological information including medication dosage, treatment duration, and adherence, we cannot fully rule out the potential confounding effects of pharmacotherapy on adolescent FA. Future research urgently needs to integrate comprehensive medication records with clinical follow-up data to more precisely evaluate the impact of psychotropic medications on FA. Finally, although this study concentrated on specific predictors, existing literature suggests that other variables, including loneliness, impulsivity, sleep quality, and family dynamics, also play a significant role in shaping dietary behaviors (54–57). Among these, sleep problems warrant particular attention. Accumulating evidence indicates that sleep disturbances can lead to impaired emotion regulation, aberrant reward sensitivity, enhanced craving, diminished executive function, and increased impulsive eating behaviors. Furthermore, sleep disruption is closely associated with depressive symptoms and problematic internet use, suggesting that it may serve as a key transdiagnostic factor linking these clinical phenotypes. Therefore, future research should adopt more comprehensive multidimensional assessment strategies and incorporate potential confounders such as sleep quality into analytical frameworks to more accurately elucidate the complex etiological network of FA.

## 5 Conclusion

In conclusion, given that adolescence is a critical period for the shaping of dietary behaviors and physical development, the dietary health of adolescents with MDD, who constitute a vulnerable population, warrants significant attention. This study revealed that approximately one-fifth of adolescents with MDD exhibited FA, driven by multiple psychosocial factors including BMI, IA, anxiety, and stress levels. However, in light of the ongoing debate regarding the nosological validity of FA, these findings should be interpreted as highlighting a prevalent cluster of maladaptive, addiction-like eating behaviors rather than confirming a definitive addictive disorder. Therefore, clinical practice should incorporate assessments of such dysregulated eating patterns into routine diagnostic and treatment protocols. Implementing targeted early intervention strategies for high-risk patients is essential to optimize treatment outcomes and improve prognosis, while future research must continue to refine the conceptualization of FA to ensure that clinical interventions are grounded in a robust and universally accepted evidence base.

## Data Availability

The raw data supporting the conclusions of this article will be made available by the authors, without undue reservation.
